# Current Perspectives on *Mycobacterium avium* Complex: Taxonomy, Epidemiology, Resistance and Genomics

**DOI:** 10.3390/ijms27135949

**Published:** 2026-07-02

**Authors:** Constança Ferreira, Paulo Gonçalves, Sónia Silva, Elsa Leclerc Duarte, Miguel Pinto, Rita Macedo

**Affiliations:** 1 National Reference Laboratory for Mycobacteria, Department of Infectious Diseases, National Institute of Health, 1649-016 Lisbon, Portugal; 2 Mediterranean Institute for Agriculture, Environment and Development (MED), Global Change and Sustainability Institute (CHANGE), Department of Veterinary Medicine, University of Évora, 7004-516 Évora, Portugal; 3 Genomics and Bioinformatics Unit, Department of Infectious Diseases, National Institute of Health, 1649-016 Lisbon, Portugal

**Keywords:** nontuberculous mycobacteria, *Mycobacterium avium* complex, epidemiology, antimicrobial resistance, genomic surveillance, One Health

## Abstract

Nontuberculous mycobacteria (NTM) are environmental opportunistic pathogens with increasing clinical relevance worldwide. Among them, the *Mycobacterium avium* complex (MAC), comprising species such as *M. avium*, *M. intracellulare*, and *M. chimaera*, is responsible for the majority of human NTM diseases. MAC causes chronic pulmonary disease and disseminated infections, particularly in immunocompromised individuals, although infections in immunocompetent hosts are increasingly reported. Despite advances in molecular diagnostics, accurate species- and subspecies-level identification remains challenging due to high genetic diversity and biased genomic databases. This limitation hampers the understanding of transmission dynamics, antimicrobial resistance patterns, and epidemiological trends. In recent years, whole-genome sequencing (WGS) has emerged as a key tool for high-resolution typing, enabling improved phylogenetic analysis, outbreak investigation, and resistance prediction. This review summarizes current knowledge on MAC taxonomy, clinical manifestations, antimicrobial resistance mechanisms, and ecological distribution, with a particular focus on the role of genomic surveillance. We highlight the need for integrated genomic frameworks to support early detection, accurate classification, and effective public health surveillance of MAC infections globally in a One Health perspective.

## 1. Introduction

The *Mycobacterium* genus was first discovered in 1874 by Armauer Hansen [1]; however, it was only in 1896 that it was formally established by Lehmann and Neumann [2]. Mycobacteria are aerobic, non-motile, and non-spore-forming microorganisms [2,3] that are widely distributed in the environment, being commonly found in water, soil, and dust [4,5,6]. Their colony morphology varies from rough to smooth and from pigmented to nonpigmented phenotypes [7]. These microorganisms possess a unique lipid-rich cell wall composed of acyl lipids, porins, arabinogalactan, peptidoglycans, and long chains of mycolic acids (60–90 carbon atoms). This structure confers hydrophobicity and low permeability, increasing resistance to chemical agents such as antimicrobials and disinfectants, as well as to adverse environmental conditions [8,9,10]. In addition, this hydrophobic cell envelope facilitates biofilm formation, further enhancing persistence in both environmental and clinical settings and resistance to antibiotics [8,9,10]. To date, more than 300 potential *Mycobacterium* species have been proposed [11].

Mycobacteria can be classified according to (i) growth rate, distinguishing rapid growers (RGM), which form colonies within 3–7 days on solid media, and slow growers (SGM), which require more than one week [12]; (ii) pigmentation, following the Runyon classification, in which SGMs are divided into photochromogens (group I), scotochromogens (group II), and nonchromogens (group III), while RGMs constitute group IV and may or may not produce pigment [13] and (iii) pathogenicity, including strict pathogens such as *M. tuberculosis*, *M. leprae*, and *M. ulcerans*, saprophytic species such as *M. gordonae*, *M. terrae*, and *M. smegmatis*, and opportunistic pathogens such as *M. xenopi*, *M. kansasii*, and the *Mycobacterium avium* complex (MAC) [14]. Based on differences in pathogenic potential, in vitro growth characteristics, and epidemiology, members of the genus *Mycobacterium* are commonly grouped into four major categories: *M. tuberculosis*, *M. leprae*, *M. ulcerans*, and the remaining species collectively referred to as nontuberculous mycobacteria (NTM) [15].

NTMs were first recognized as opportunistic pathogens in the mid-1950s [16]. However, their clinical relevance became particularly evident following the identification of MAC as a major cause of disseminated infections in patients with acquired immunodeficiency syndrome (AIDS) [17]. Since then, NTM pulmonary infections have been increasingly recognized and are now considered an emerging public health concern in many regions worldwide [18]. In the NTM group, the majority of species are environmental opportunistic pathogens widely distributed in soil, water, and air. The prevalence of NTM infections has globally increased since 2000 [19,20,21], a trend attributed to population aging, improved diagnostic methodologies, and a growing number of immunocompromised individuals [17,22]. However, the true clinical burden of NTM disease remains difficult to estimate, largely due to the challenge of distinguishing colonization from active infection. Additionally, as NTM disease is not subject to mandatory notification in many settings, cases are frequently underreported or misdiagnosed as *M. tuberculosis* infections. Consequently, epidemiological estimates often rely on laboratory isolation data rather than clinically confirmed disease incidence [23,24]. This limitation underscores the need for standardized diagnostic criteria and the integration of molecular and genomic surveillance strategies to improve case definition and epidemiological accuracy.

Among the different NTM species, those belonging to MAC are the most clinically significant. In immunocompromised individuals, MAC infections are associated with severe disease, poor prognosis, and increased mortality. Moreover, recent studies have also reported a rising number of infections in immunocompetent hosts. Overall, over the last decade, MAC species have accounted for the majority of clinically relevant NTM infections worldwide [25,26,27]. In this context, this review aims to summarize the current knowledge regarding MAC epidemiology, taxonomy, antimicrobial resistance, and genomic surveillance, highlighting the need for implementation of integrated molecular surveillance systems.

## 2. *Mycobacterium avium* Complex

The first MAC-associated disease was reported in the 1890s [27], although at the time it was only recognized as a member of the genus *Mycobacterium*. In 1933, MAC species were finally considered potentially pathogenic to humans, following the isolation of *M. avium* from birds exhibiting cavitary disease resembling tuberculosis [28]. The first documented case of human pulmonary infection caused by *M. avium* was reported in 1943 [27]. In the 1990s, the MAC was commonly referred to as *Mycobacterium avium-intracellulare* (MAI), as it originally included two main species: *M. avium* and *M. intracellulare* [29,30]. With the advances in molecular diagnostic techniques, particularly gene sequencing, and later whole-genome sequencing (WGS), the taxonomic structure of MAC has expanded considerably, with multiple additional species being incorporated into the complex. These taxonomic refinements reflect the increasing resolution provided by molecular and genomic approaches, which continue to reshape the understanding of MAC diversity. *M. avium* has four recognized subspecies: *M. avium* subsp. *avium* (MAA), *M. avium* subsp. *paratuberculosis* (MAP), *M. avium* subsp. *silvaticum* (MAS) [31], and *M. avium* subsp. *hominissuis* (MAH) [32]. Although genetically closely related, these subspecies differ in host tropism, pathogenicity, and ecological niche. MAA is responsible for the majority of avian mycobacteriosis [31], while MAP is the causative agent of Johne’s disease in ruminants [33] and has been investigated for potential associations with human diseases such as type 1 diabetes, multiple sclerosis, and Crohn’s disease [34,35,36,37,38,39]. MAS is mainly associated with disease in wood pigeons [32], and MAH is the most clinically relevant subspecies in humans, accounting for the majority of human MAC infections and displaying the highest genetic diversity within the *M. avium* subspecies complex [40]. Unlike the relatively well-defined taxonomy of *M. avium* subspecies, the classification of *M. intracellulare*-related organisms remains less stable, with ongoing re-evaluation of species boundaries [41]. Historically, in 1992, only *M. avium* and *M. intracellulare* were recognized as distinct species within MAC, alongside genetically distinct variants (MAC-A to MAC-H) that differed in the internal transcribed spacer (ITS) sequences [41]; however, these represented informal genetic variants rather than formally recognized species. In 2004, Tortoli et al. proposed elevation of one of these variants to species level as *M. chimaera* [42]. In 2018, van Ingen and colleagues proposed the inclusion of ten additional species to the complex: *M. colombiense*, *M. arosiense*, *M. marseillense*, *M. lepraemurium*, *M. paraintracellulare*, *M. bouchedurhonense*, *M. vulneris*, *M. timonense*, *M. yongonense*, and *M. chimaera* [43]; however, subsequent genomic studies have suggested further taxonomic refinement, including the reclassification of *M. yongonense* and *M. chimaera* within the *M. intracellulare* lineage, and the proposal that *M. intracellulare* subsp. *yongonense* and *M. intracellulare* subsp. *chimaera* may represent highly closely related taxa within the same subspecies group [44]. More recently, Tortoli et al. also highlighted the high genomic similarity between *M. intracellulare* subsp. *intracellulare* and *M. paraintracellulare*, suggesting that these taxa may not represent clearly distinct subspecies and could potentially be consolidated within a single subspecific lineage [40]; however, such proposals remain under discussion, reflecting ongoing uncertainty in MAC taxonomy.

As the taxonomy of the MAC remains subject to revision and alternative classifications exist for some taxa, with some authors preferring to use *M. chimaera* as a species and others as a subspecies, this review will follow the nomenclature proposed by van Ingen et al. (2018) [43].

## 3. Ecological Niche and Transmission Pathways

**Figure 1:**
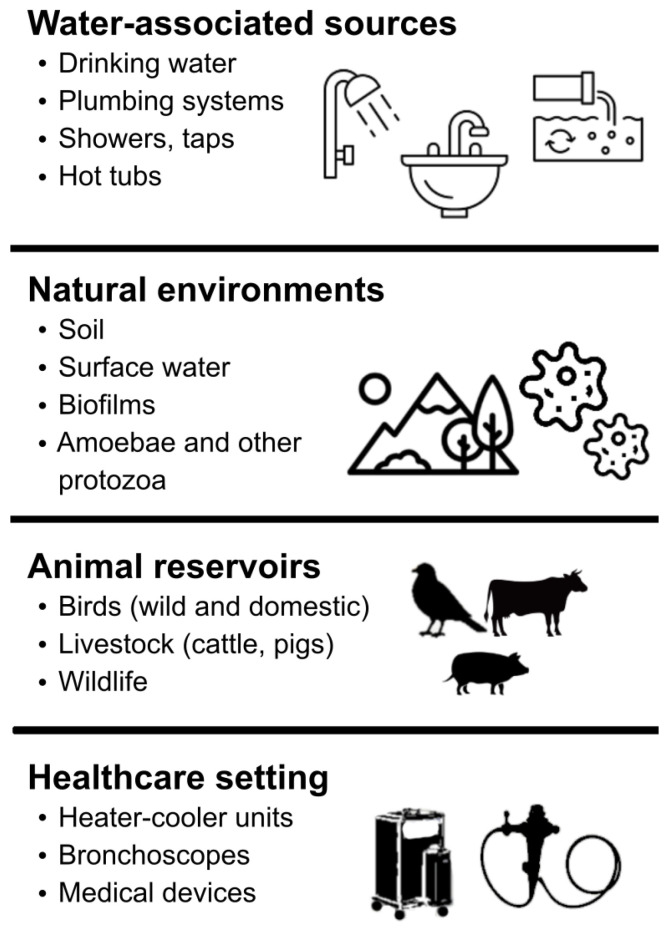
*Mycobacterium avium* complex environmental reservoirs.

Species from MAC are ubiquitous in the environment, and their main reservoirs are water systems, soil, and vegetation [6] (Figure 1). MAC members exhibit natural resistance to disinfectants and are able to survive under low-pH conditions, which contributes to their environmental persistence [45,46]. The main routes of transmission are believed to occur through ingestion or inhalation of contaminated water and aerosols [6,47], as MAC members are highly resistant and can persist in water distribution systems, frequently colonizing showerheads and faucets. These bacteria can exist as free-living organisms or within biofilms, and association with free-living amoebas has also been described [6,48]. Such interactions may favor the selection of traits associated with virulence and persistence, further contributing to their survival in water systems [48,49]. Their persistence and proliferation in these environments are facilitated by their hydrophobic cell surface and oligotrophic capacity, which allow adhesion to surfaces and biofilm formation [6,50]. Moreover, due to their microaerophilic metabolism, MAC species can establish themselves at the air–water interface and become aerosolized from showers, taps, and recreational water facilities such as swimming pools. The lipid-rich cell wall of MAC species also contributes to resistance against chemical disinfectants, including chlorine and ozone, commonly used in water treatment systems [49]. Consequently, water disinfection processes may inadvertently select for mycobacteria by eliminating competing microorganisms while allowing MAC species to persist [49]. These characteristics reinforce the importance of environmental reservoirs in the epidemiology and transmission dynamics of MAC infections.

Keen et al. demonstrated that pangenomes of isolates recovered from similar environmental niches exhibit high genomic similarity, suggesting that accessory genes could play an important role in adaptation to specific environments [50]. These genes, which constitute the variable component of the pangenome, may confer selective advantages such as enhanced biofilm persistence, tolerance to adverse environmental conditions, and improved host colonization capacity. In the same study, isolates obtained from different sources—including human pulmonary and disseminated infections, animals, and environmental samples—were shown to be genomically distinct. Comparative analyses of core and accessory genomes, gene content, and mobile genetic elements suggested niche-specific adaptation rather than random environmental distribution [50]. These findings further support the growing evidence that MAC populations are highly structured according to ecological niche and host association. Importantly, the genomic variability associated with accessory elements highlights the limitations of conventional phenotypic approaches for strain differentiation, as relevant genomic differences may not be reflected in observable phenotypes.

MAC species are also commonly found in health care facilities, where they can colonize medical equipment such as bronchoscopes and the water used to clean them, thus contributing to nosocomial infections. Recently, several cases of infections by *M. chimaera* in patients subjected to open heart surgery were reported and later associated with contaminated heater-cooler units [51,52,53]. These cases often occur after long incubation periods and are usually associated with heart infections and endocarditis, both with high mortality rates [53]. Even though some MAC species have already been isolated from vegetables and fruits [54], dairy [55,56,57], and meat products [54,55,57,58], their zoonotic potential is not yet confirmed. Still, several studies have highlighted the occurrence of MAC infections in animal reservoirs (mainly subsp. *M. paratuberculosis*) such as birds, livestock, pigs, and wildlife species (Figure 1), reinforcing the relevance of these organisms and the close interaction between animal, environmental, and human health [32,33,34,35,36,37,38,39,40]. The broad distribution of MAC members across different environmental niches and animal hosts suggests that animals may contribute to the dissemination and maintenance of these microorganisms in shared ecosystems. Additionally, the detection of MAC organisms in animal-derived food products and environmental sources further supports the need for integrated surveillance approaches addressing the roles of animals, humans, and the environment in MAC epidemiology [50,55,56,57,58]. Unlike other NTM, there are no documented cases of person-to-person transmission, and, therefore, it is believed that transmission occurs through exposure to contaminated environmental/animal sources [59].

## 4. Infection, Clinical Manifestations, and Treatment

Despite frequent environmental exposure, MAC disease remains relatively uncommon, suggesting that host immune defenses are generally effective in preventing infection. Individuals who develop the disease are thought to possess specific susceptibility factors; however, the underlying mechanisms enabling infection establishment are not yet fully understood [20,60]. Phenotypic characteristics, particularly colony morphotype, play an important role in MAC pathogenicity. Rough morphotypes exhibit altered or absent glycopeptidolipid (GPL) expression compared to smooth variants [7,61,62]. GPLs are involved in immune modulation, surface interactions, and biofilm formation, which contribute to host colonization, bronchial epithelial invasion, and persistence within the respiratory tract [63]. Smooth variants, particularly the smooth transparent morphotype, have traditionally been associated with enhanced colonization, immune system evasion, and intracellular survival, properties that have been linked to the presence of surface GPLs [62,63]. Still, the relationship between colony morphology and virulence remains complex, as some studies show that rough variants exhibit enhanced inflammatory potential and high virulence in both macrophage and murine infection models [7,64].

**Figure 2:**
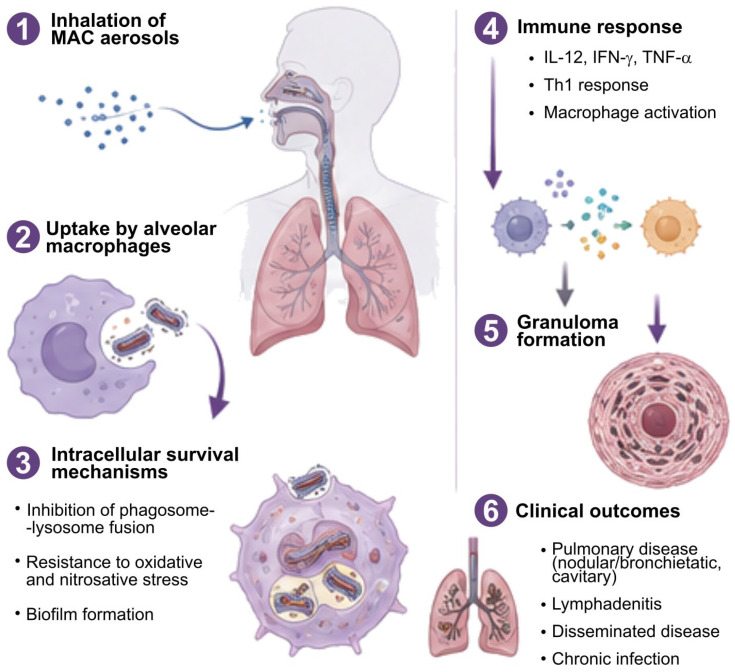
*Mycobacterium avium* complex host infection and pathogenesis in pulmonary disease. MAC—*Mycobacterium avium* complex; IL-12—interleukin-12; IFN-γ—interferon-gamma; TNF-α—tumor necrosis factor-alpha; Th1—T helper 1 cell.

In addition, MAC species can survive acidic conditions in the gastrointestinal tract, enabling passage through the gastric barrier and potential dissemination following ingestion [46,65,66]. Following infection, phagocytosed mycobacteria trigger complex host immune responses [67]. Key cytokines, including interleukin-12 (IL-12), interferon-gamma (IFN-γ), and tumor necrosis factor-alpha (TNF-α), play crucial roles in regulating the antimycobacterial immune response [67,68]. IL-12 promotes activation of natural killer cells and proliferation of T-lymphocytes [69,70], while IFN-γ and TNF-α enhance macrophage activation and intracellular killing capacity. Granulocyte-macrophage colony-stimulating factor further contributes to macrophage functional activation and control of intracellular replication [65,70,71] (Figure 2).

Clinical manifestations vary according to host status and infecting species (Table 1). Pulmonary disease is the most common presentation and is classically divided into nodular bronchiectatic disease (non-cavitary or cavitary) and fibrocavitary disease [72]. Evidence suggests species-specific associations, with *M. intracellulare* more frequently linked to cavitary disease, whereas nodular bronchiectatic disease may be caused by multiple MAC species [27,64,72,73].

Disseminated MAC disease occurs predominantly in severe immunocompromised individuals, such as those with AIDS or organ transplant recipients, for which *M. avium* is the most frequently isolated species [74,75,76]. Dissemination typically occurs via hematogenous spread, with involvement of multiple organs, including lymph nodes, spleen, liver, intestines, and bone marrow. In immunocompetent individuals, pulmonary MAC disease is usually chronic and slowly progressive, presenting as pneumonia, nodular bronchiectasis, and fibrocavitary disease [77]. In this type of infection, following inhalation of aerosolized bacteria, alveolar macrophages serve as primary host cells. The lipid-rich mycobacterial cell wall contributes to resistance against intracellular killing by inhibiting phagosome–lysosome fusion, enabling intracellular survival and long-term persistence within lung tissue [68]. Persistent infection induces a granulomatous immune response characterized by recruitment of immune cells surrounding infected macrophages and ultimately leading to granuloma formation [68].

Cervical lymphadenitis caused by MAC is predominantly observed in children under five years of age [78]. It presents as chronic, usually unilateral lymph node enlargement, often without systemic symptoms. Diagnosis relies on microbiological confirmation from lymph node samples. While spontaneous regression can occur, progression with liquefaction and persistent drainage is common [78]. Still, clinical presentation of MAC infections is generally nonspecific and may include low-grade fever, fatigue, weight loss, chronic cough, dyspnea, and night sweats [15]. This lack of specificity often leads to delayed diagnosis and inappropriate initial treatment. Radiologically, MAC pulmonary disease may mimic tuberculosis or other chronic lung infections, often presenting with nodules, bronchiectasis, or cavitary lesions [79].

Historically, NTM infections were treated using standard anti-tuberculosis regimens; however, their limited efficacy soon became clear [80,81,82]. The introduction of macrolides significantly improved treatment outcomes, although monotherapy is strongly discouraged in order to minimize the risk of resistance [81]. Current MAC treatment selection depends not only on species identification and susceptibility profiles, reinforcing the importance of precise microbiological diagnosis, but also on clinical and radiographic phenotype of the disease, namely cavitary or nodular bronchiectatic patterns. Guidelines jointly developed by the American Thoracic Society (ATS), European Respiratory Society (ERS), European Society of Clinical Microbiology and Infectious Diseases (ESCMID), and Infectious Diseases Society of America (IDSA) recommend multidrug therapy, typically including a macrolide, ethambutol, and a rifamycin, for at least 12 months after culture conversion [81]. In severe cases, aminoglycosides, such as amikacin, may be added. For patients with nodular bronchiectatic disease without cavitation, which represents the most common phenotype, an intermittent regimen—three times weekly—consisting of a macrolide (azithromycin or clarithromycin), ethambutol, and a rifamycin is recommended. In contrast, patients with progressive disease or higher bacterial burden should receive daily therapy. In fibrocavitary disease, which is typically more aggressive and associated with higher mycobacterial load and worse outcomes, daily therapy—seven days per week—is the recommended treatment. For cavitary, extensive nodular/bronchiectatic disease, amikacin or streptomycin could be given three times per week. Susceptibility testing is recommended to guide therapy, particularly for macrolides, aminoglycosides, and fluoroquinolones [81]. Particularly, in macrolide-resistant MAC disease, prognosis is significantly worse, and treatment options are limited. Management typically involves a backbone of ethambutol and a rifamycin combined with parenteral or inhaled amikacin, with consideration of additional agents such as clofazimine or linezolid in selected cases [81]. These patients should be managed in specialist centers due to high rates of treatment failure [81]. Refractory disease, defined by persistent culture positivity after approximately six months of appropriate therapy, requires a daily treatment regimen with inhaled liposomal amikacin, which is commonly added in this setting [81]. Treatment outcomes for MAC disease remain variable and are generally less favorable than those observed for other NTM infections [83]. Prolonged therapy duration, drug toxicity, and complex regimens contribute to reduced adherence and therapeutic failure [81,83]. Emerging antimicrobial resistance, particularly to macrolides and aminoglycosides, further complicates management and emphasizes the need for continuous surveillance of resistance patterns [83]. While drug susceptibility testing (DST) remains the clinical standard for macrolides, like clarithromycin, and aminoglycosides, like amikacin, for other antibiotics, like ethambutol or rifampicin, the correlation between in vitro results and clinical outcomes is inconsistent.

As the genetic and adaptive mechanisms underlying resistance remain to be fully disclosed, the integration of molecular diagnostics and WGS is increasingly recognized as essential for improving the prediction of resistance towards guiding personalized therapy [81]. Novel therapeutic strategies, including optimized combination regimens and genome-informed approaches based on WGS data, are under investigation to improve treatment outcomes and limit resistance emergence [81,83]. Given the species-dependent variability in drug susceptibility and clinical outcomes, accurate identification at the species and subspecies level remains critical for effective clinical management and improved patient prognosis [29,61].

## 5. Epidemiology

Several studies, conducted over the last decade, have attempted to elucidate the global distribution of NTM disease [24,25,26]. In 2013, Hoefsloot et al. [24] and, in 2019, Farnia et al. [84] conducted worldwide studies that showed global geographic diversity of NTM species through the analysis of clinical and environmental isolates [24,84]. These studies identified MAC species as the main etiological agents responsible for most clinical cases and suggested an association between geographic distribution and different MAC species [24]. More recent multi-country surveillance and population-based studies have generally reported MAC as the most frequently identified NTM group across most regions of the world, although the heterogeneity of the studies limits linear cross-data comparisons [23,85,86,87,88,89,90,91,92]. However, in several contemporary datasets, MAC typically accounts for approximately 40–80% of all clinical NTM isolates and NTM cases, although its reported frequency, again, varies according to study design and surveillance methodology [20,24,85,93,94]. Laboratory-based studies from Europe and East Asia consistently report MAC as the leading species among cultured isolates [24,26,93], whereas large national registry-based studies, particularly in South Korea, confirm a growing burden of NTM disease but often lack detailed species-level resolution [95]. To better illustrate these global differences, Table 2 summarizes key epidemiological studies reporting MAC distribution across different regions. Many epidemiological surveys reported isolates as *M. avium* or *M. intracellulare* without subspecies- or lineage-level characterization and often did not specify the taxonomic framework or nomenclature adopted. Therefore, throughout this section and in Table 2, the nomenclature reported in the original publications has been retained to ensure consistency and avoid retrospective taxonomic reassignment of isolates for which more detailed characterization was not available.

As shown in Table 2, MAC was generally reported as the most frequently identified NTM group, both in high-income and middle-income settings, regardless of the studies’ design, denominator, case definition, dataset broadness and size, and surveillance strategy. Notably, *M. avium* and *M. intracellulare* remain the most frequently isolated species within the complex, although their relative proportions vary geographically [20,24,93]. These differences are thought to reflect a combination of environmental exposure, host susceptibility, and local diagnostic practices rather than true absence or presence of specific species [20,93]. Epidemiological studies indicate geographic differences in the distribution of NTM, with MAC consistently identified as one of the most frequently isolated groups across diverse continents, including Asia, Europe, and North America, although its reported impact varies by region [24,26,93]. Available evidence suggests a predominant association of MAC with pulmonary disease [15,20], while extrapulmonary manifestations, such as skin and soft tissue infections, disseminated disease, and lymph node involvement, occur less frequently and are mainly observed in immunocompromised individuals [66,75,78].

The infection primarily affects older adults (>55 years old), individuals with underlying structural lung diseases, such as chronic obstructive pulmonary disease and bronchiectasis, and immunosuppressed patients [20,77]. Nevertheless, the true incidence and prevalence of MAC-associated disease remain difficult to assess. Substantial heterogeneity in diagnostic criteria, surveillance systems, and laboratory methodologies contributes to variability in reported epidemiological data, limiting direct comparisons across regions [14,20,93]. In particular, differences between laboratory-based studies, reporting species-level data, and population-based registry studies, without microbiological confirmation, further complicate global comparisons. Additionally, there is an ongoing challenge in distinguishing true infection from airway colonization, which may lead to both underestimation and overestimation of disease burden in different settings [47,77]. As a result, epidemiological assessments of MAC infection remain complex, and its overall public health impact is likely influenced by both methodological limitations and differences in diagnostic capacity across regions [14,20,93].

Overall, recent evidence consistently identifies MAC as the most frequently reported NTM group across many worldwide studies, although direct comparisons between studies remain limited due to the significant variability in surveillance systems, laboratory capacity, and species identification methods [20,93]. In order to circumvent the heterogeneity of study design and datasets, the development of a robust and standardized approach, contributing to inter-laboratory and inter-sector results comparability, would be beneficial towards improving MAC surveillance. The limited availability of global epidemiological data also reinforces the need for more standardized surveillance systems incorporating robust species-level identification and geographically representative sampling to better characterize the true burden of MAC infections globally [14,93].

## 6. Diagnosis

As previously mentioned, MAC species are ubiquitously present in the environment, and their frequent laboratory isolation from clinical respiratory specimens may not confirm active disease. As such, the differentiation between disease and colonization requires the integration of radiological, clinical, and microbiological criteria, as outlined in the ATS and the Infectious Disease Society of America [77]. Briefly, to classify a case as “disease-associated”, at least two positive cultures of the same species, isolated from respiratory specimens collected in different time periods (e.g., one week apart), are required. Conversely, for other biological samples, such as biopsies or specimens collected using invasive procedures (bronchoalveolar lavage or bronchial washes), a single positive MAC isolation is sufficient to confirm suspected disease (i.e., with compatible symptoms and histopathological findings). Radiological criteria are based on chest radiography showing nodular or cavitary opacities, or computed tomography findings demonstrating bronchiectasis with multiple nodules [77,81].

**Figure 3:**
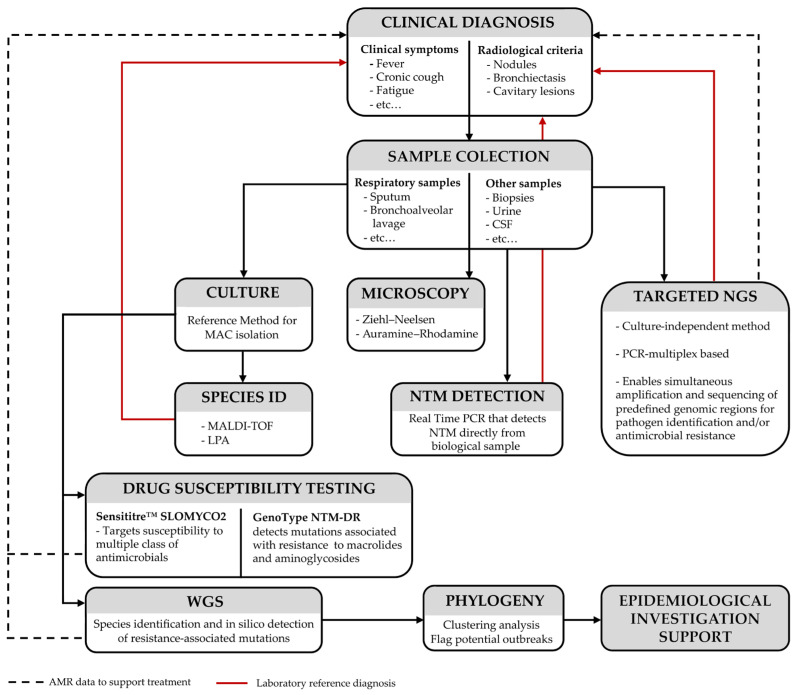
Integrated workflow for the laboratory diagnostics and genomic characterization of MAC to support clinical diagnosis and epidemiological investigation. MALDI-TOF: Matrix-Assisted Laser Desorption/Ionization Time-of-Flight; LPA: line-probe assays; AMR: antimicrobial resistance; CSF: Cerebrospinal Fluid; ID: identification; WGS: whole-genome sequencing.

Traditional diagnostic methods (Figure 3) include culture, which primarily distinguishes between slow- and fast-growing mycobacteria; microscopy, where clinical samples are stained using methods such as Ziehl–Neelsen or Auramine–Rhodamine; and molecular testing and mass spectrometry (MALDI-TOF), allowing species-level identification [96,97,98]. Common commercial kits include GenoType Mycobacterium CM [99], which targets the most clinically relevant and frequently encountered species, GenoType Mycobacterium AS [100], which expands detection to less common species, and Inno-LiPA Mycobacteria [101], an alternative platform based on a similar principle but less widely used today. In addition, some kits, such as GenoType NTM-DR [102], can detect mutations associated with antimicrobial resistance, particularly to macrolides and aminoglycosides, enabling both species identification and resistance profiling. These line-probe assays (LPA) are generally faster than sequencing-based approaches but have limited resolution for rare or newly described species not included in their probe panels [103]. Species identification is mainly based on nucleic acid sequencing of conserved genomic regions. Most common targets include *16S rDNA*, *hsp65*, and the *16S–23S* ITS regions, which are particularly useful for species-level differentiation due to their variability, but other genes such as *gyrA*, *gyrB*, *sodA*, *secA1*, and *rpoB* have also been used [104].

Recent reviews have highlighted the potential application of Clustered Regularly Interspaced Short Palindromic Repeat-associated proteins (CRISPR- CAS) for rapid identification of nontuberculous mycobacteria, although most approaches remain experimental and require further clinical validation [105]. This technique uses guide RNA to recognize target DNA sequences, activating Cas proteins that cleave reporter molecules and produce a fluorescent signal for detection [105]. Another recently developed technology, MyTRACK, is a portable system that combines Recombinase Polymerase Amplification with CRISPR-Cas12a to detect and differentiate *M. tuberculosis* complex and NTM [105]. In 2025, Compiro et al. compared the performance of MyTRACK with LPAs for mycobacterial detection, showing detection of 1–100 copies per reaction and demonstrated 100% specificity and 92.59–100% sensitivity, with data consistent with culture and LPA methods [106].

DST using broth microdilution (BMD) remains the definitive reference standard for antimicrobial resistance predictions [107]. The minimum inhibitory concentrations (MICs) are determined using standardized commercial platforms such as the Sensititre™ SLOMYCO2 panel [108], targeting susceptibility to macrolides, like clarithromycin, and to amikacin [81,107]. MAC needs an extended incubation period—7 to 14 days—to ensure metabolic stability and accurate MIC determination [107]. Phenotypic BMD captures the cumulative physiological response of the isolate, providing the key breakpoints to guide complex multidrug regimens [81].

Still, despite their clinical utility, these conventional approaches often exhibit limited discriminatory power, which may compromise accurate species-level identification and restrict the completeness of antimicrobial resistance profiling, particularly in the context of rare, newly described, or phylogenetically closely related NTM species [107]. In addition, phenotypic methods such as BMD, while considered the reference standard, are inherently time-consuming and may not reflect potential underlying genomic determinants of resistance in an infecting population, especially in cases where inducible or low-level resistance mechanisms are involved [107]. As a result, there is an increasing need for more advanced molecular approaches capable of providing higher-resolution and more comprehensive genetic information. In this context, next-generation sequencing (NGS) has emerged as a powerful tool that enables WGS analysis, offering improved accuracy in species identification and enhanced detection of resistance-associated mutations. It has become possible to overcome several of these intrinsic limitations by enabling high-throughput, culture-independent, and genome-wide analyses that provide unprecedented depth and resolution in the diagnosis of NTM infections, including those caused by the MAC [109]. NGS-based approaches allow for the simultaneous identification of complex microbial communities and the comprehensive detection of antimicrobial resistance genes, including rare, emerging, or previously uncharacterized determinants that may be clinically relevant in MAC infections [81,110]. In addition, these methodologies facilitate the reconstruction of genomic contexts such as plasmids, transposons, and other mobile genetic elements associated with horizontal gene transfer, which may contribute to the dissemination of resistance traits within heterogeneous mycobacterial populations [107].

## 7. Mechanisms of Antimicrobial Resistance

Like most NTM species, antimicrobial resistance in MAC is mediated by several intrinsic and acquired mechanisms that collectively contribute to reduced susceptibility to multiple classes of antibiotics [77,111]. These mechanisms include structural and physiological barriers, target site modifications, active drug efflux pumps, enzymatic inactivation, and biofilm-associated tolerance, which may act independently or in combination.

Intrinsic resistance is mainly associated with the lipid-rich and highly hydrophobic mycobacterial cell envelope, which restricts antibiotic penetration and reduces drug susceptibility [77,112]. Additional intrinsic mechanisms include low cell envelope permeability [112] and production of β-lactamases capable of enzymatic drug degradation; however, the precise contribution of β-lactamases to β-lactam resistance in MAC remains unclear, as resistance is considered multifactorial and also influenced by other intrinsic defense mechanisms. Importantly, recent evidence shows that β-lactam combinations can exhibit bactericidal activity against MAC, supporting the role of target redundancy in intrinsic resistance rather than β-lactamase-mediated hydrolysis alone [113].

Biofilm formation is an important tolerance-associated mechanism in NTMs that reduces antimicrobial efficacy and promotes bacterial persistence by acting as a physical barrier that limits antimicrobial penetration [48]. These can also contain metabolically less active bacterial populations that exhibit increased phenotypic tolerance to antimicrobial agents without necessarily carrying resistance-conferring mutations. In NTMs, biofilm environments may also promote horizontal gene transfer between the biofilm-forming bacteria that could contribute to the spread of drug resistance mechanisms [114]. Recent genomic studies have suggested that plasmids are more common and diverse in MAC than previously reported. Although their contribution to antimicrobial resistance remains incompletely understood, plasmids may contribute to genetic adaptation and could potentially influence antimicrobial susceptibility [115].

Acquired resistance can develop through mutations affecting antimicrobial target sites, reducing drug binding and therapeutic activity [116], often linked to the independent emergence of multidrug resistance [117]. Mutations in *16S rRNA* (*rrs*) and *23S rRNA* (*rrl*) genes confer resistance to aminoglycosides and macrolides, respectively, while mutations in the *rpoB* gene, encoding the β-subunit of RNA polymerase, result in a blockage of RNA synthesis conferring resistance to rifamycins [112,118,119,120,121]. Mutations in the quinolone resistance-determining region (QRDR) of *gyrA* and *gyrB* could alter the DNA gyrase targets, potentially reducing fluoroquinolone susceptibility; however, their contribution to fluoroquinolone resistance in MAC remains uncertain, as some studies have reported associations between mutations in these genes and elevated MICs, whereas others failed to identify resistance-associated mutations in fluoroquinolone-resistant isolates [122,123,124].

Other important resistance mechanisms include efflux pump-mediated drug extrusion, inducible tolerance systems, and enzymatic drug modification, all of which contribute to reduced antimicrobial susceptibility and persistence during therapy. Several efflux systems have already been identified in MAC [104,125,126], regulating the accumulation of antimicrobials inside the cell, and being also part of the resistance-nodulation-division (RND)-like transport systems [104]. For example, in the *mmpL* gene family, MmpL5/MmpS5 is involved in expelling lipophilic compounds and antibiotics and has been associated with resistance to bedaquiline and clofazimine [126]. The genes *MAV_3306*, *MAV_1406*, and *MAV_1695* (Genbank accession number NC_008595.1) have been associated with an inducible efflux system whose expression increases antibiotic tolerance while promoting active drug extrusion, particularly for macrolides [126]. In parallel, the *EmbAB* system is involved in cell envelope lipid transport and organization, which may reduce intracellular drug accumulation [127]. Together, these mechanisms contribute to antimicrobial tolerance and decreased susceptibility in MACs. Prolonged antibiotic exposure may also promote the selection of resistant subpopulations, particularly during long-term multidrug treatment regimens. In addition, the slow growth rate, intracellular survival, and ability to persist under stressful environmental conditions further impact treatment. As such, MAC infections may be particularly challenging to eradicate, highlighting the importance of susceptibility testing and optimized combination treatment regimens. The resistance mechanisms associated with clinically relevant antimicrobials used against MAC are summarized in Table 3.

Although several genes and resistance mechanisms have been identified in MACs, a comprehensive global overview with association to resistance phenotypes, like the one compiled for *M. tuberculosis* [130], is still lacking. Given the complexity of antimicrobial resistance mechanisms in MAC and the limitations of conventional diagnostic approaches, there is a growing need for more comprehensive tools to characterize the full resistome. In this context, NGS offers a high-resolution approach to better survey resistance determinants and their association with relevant antimicrobial susceptibility phenotypes.

## 8. MAC Genomics

MAC genomes display high GC content, typical of mycobacteria, and large genome sizes ranging between 4.5 and 5.5 Mbp, with substantial variability across their species. In fact, comparative genomic analyses performed after the availability of the first complete MAC reference genomes (*M. avium* strain 104 and MAP strain K-10 [131] revealed extensive sequence variation between human- and animal-associated isolates [132,133,134]. Studies have reported that MAC species share a small core-genome size, and their diverse accessory genome reflects the divergent adaptive strategies across lineages [50,135,136,137].

A prominent feature of MAC genomes is the abundance of repetitive sequences and insertion elements (IS elements), which contribute to genomic plasticity through duplications, structural rearrangements, and recombination events, while also playing a role as epidemiological markers [138,139]. Among the most relevant IS elements in MACs are *IS1245*, *IS1311*, and *IS901*, each with distinct distribution patterns and functional significance. *IS1245* is predominantly associated with MAH, where it is typically present in multiple copies, generating highly polymorphic restriction-fragment length polymorphisms (RFLP) patterns that have historically been used for strain differentiation [140,141]. In avian-associated isolates, *IS1245* is often present in lower copy numbers, while it is generally absent in MAP and *M. intracellulare*, making its absence a useful discriminatory feature for this subspecies [139,142]. Although more widely distributed across MAC, *IS1311* displays enough polymorphisms to enable MAP lineage distinction, particularly the so-called S-type and C-type strains [139,143]. In contrast, *IS901* is strongly associated with avian-adapted lineages of *M. avium* and is considered a marker for bird-associated strains, being largely absent in human and porcine isolates [144,145]. Its presence therefore represents a key marker of host specificity and adaptation within the complex [146].

Genome-wide single-nucleotide polymorphism (SNP) analysis has become a fundamental approach for high-resolution phylogenetics and epidemiological surveillance, enabling discrimination between closely related strains, identification of transmission clusters, and investigation of microevolutionary events occurring during host adaptation and infection. In this context, WGS has emerged as a powerful tool, leveraging the analysis of entire genomes to provide the highest possible resolution for species and subspecies differentiation, enabling clear discrimination among closely related taxa and the detection of genetic clusters with epidemiological relevance [147,148,149]. This approach enhances our understanding of the genetic diversity of circulating strains from clinical, environmental, and animal sources with the potential to support surveillance in a One Health approach. Moreover, it offers insights into transmission dynamics and potential in vivo adaptation mechanisms during infection (including antimicrobial resistance), which are critical for public health and clinical management, respectively [134,148,149]. Unlike earlier approaches based on single genetic markers, WGS allows the reconstruction of robust phylogenetic relationships and the detection of fine-scale genomic variation across entire genomes. This has revealed a highly stratified population structure within MACs, characterized by pronounced differences in genetic diversity and evolutionary dynamics between lineages [136,147]. For instance, a study involving over 1200 isolates of *M. avium* subspecies was conducted to reconstruct phylogeny and identify specific genomic characteristics. The results confirmed that the subspecies MAP, MAA, and MAS belong to the species *Mycobacterium avium* and exhibit distinct differences. It was observed that MAH possesses a large accessory genome and a small core genome, forming heterogeneous clades. MAP showed a highly conserved core genome and limited pangenome diversity, which may reflect host-adapted evolution [136]. As WGS becomes more accessible, genomic data also show the existence of potential strains that phylogenetically deviate from known subspecies identification, suggesting that the overall circulating MAC diversity is yet to be fully disclosed [137]. Additionally, MAC comparative genomic analyses based on SNP distances have enabled the identification of genetically related clusters and the investigation of potential transmission events in both environmental and clinical settings. In particular, genomic studies of *M. chimaera* outbreaks associated with contaminated heater-cooler units demonstrated the utility of WGS for source tracking, revealing highly similar genomes between patient and environmental isolates and supporting the hypothesis of contamination during device manufacturing [51]. Consistent with this, recent genomic epidemiology studies have identified genetically related MAC isolates across geographically distant regions, suggesting the existence of globally distributed lineages linked to shared environmental reservoirs or nosocomial infection, rather than direct human-to-human transmission [50,136,146,150,151]. More recent multinational genomic surveys have also highlighted the broad geographic distribution of MAC lineages and the contribution of mobile genetic elements to genomic diversification. In one European WGS study involving more than 600 clinical isolates, phylogenetic analyses identified trans-European clusters across three MAC species, *M. chimaera*, *M. avium,* and *M. intracellulare* [147]. Furthermore, in *M. intracellulare*, the plasmid prevalence observed was low, being present in only about 19% of the isolates. However, plasmid prediction analyses revealed an exceptionally high prevalence of plasmids, 98%, among *M. chimaera* isolates [147]. Another study highlights the same trend, with nearly all *M. chimaera* strains analyzed carrying multiple plasmids, with considerable variability in plasmid composition even within the same subspecies, while only a few *M. intracellulare* isolates carried plasmids [152]. These studies support the hypothesis that horizontal gene transfer may contribute to adaptation, virulence, and lineage diversification within this species; however, additional studies are needed to understand the impact of these plasmids on antibiotic resistance [147,152]. Collectively, these studies demonstrate how WGS-based comparative genomics has transformed the understanding of MAC evolution, transmission, and population structure. Still, as MAC is composed of multiple subspecies with overlapping ecological and clinical niches, WGS is applied unevenly across subspecies and sources, creating a bias in public genomic databases (and, consequently, genomic diversity), hindering the development of a robust and universal typing scheme, and limiting our ability to understand transmission patterns and subspecies-level diversity.

Gene-by-gene approaches—i.e., whole-genome (wg-) or core-genome (cg-) multi-locus sequence typing (MLST)—are being developed for MAC and used as a means to promote large-scale analysis in the context of surveillance [153], as they enable standardized large-scale comparisons with improved discriminatory power when compared with conventional typing methodologies [154]. These approaches facilitate cluster detection, comparative analyses, and high-resolution strain classification across geographically distinct collections, supporting their application in recurrence analysis, population structure, and outbreak investigation studies. Recent studies have also highlighted the clinical relevance of integrating genomic typing into MAC management, particularly for distinguishing relapse from reinfection and for improving epidemiological interpretation of recurrent MAC pulmonary disease [154]. Moreover, wg/cgMLST approaches could also be particularly relevant in disclosing transmission events in complex cases. For instance, *M. avium* subsp. *hominissuis*, which exhibits extensive genomic diversity and homologous recombination, leading to mosaic genome structures and resulting in complex SNP-based phylogenetic reconstructions, would clearly benefit from a gene-by-gene analysis that can mitigate the effects of genome-scale recombination events [155]. In contrast, *M. chimaera* displays a comparatively more clonal population structure associated with well-defined outbreak lineages and clearer genomic clustering patterns [147]. Genome-scale analyses have further demonstrated substantial interspecies and inter-subspecies diversity within MAC, as well as uneven representation of species, subspecies, and environmental sources in public genomic databases [137,156]. Collectively, these findings reinforce the need for standardized and broadly representative genomic frameworks to improve MAC surveillance, epidemiological inference, and our understanding of transmission dynamics and subspecies-level diversity. One such framework could rely on a cgMLST typing scheme targeting all MAC species in combination with an accessory genome MLST scheme (e.g., a wgMLST) for increased species resolution. These dynamic frameworks have already been successfully applied, for example, in the field of food- and water-borne diseases [157] and in *M. tuberculosis* [158]. Briefly, this approach would rely on a starting MAC cgMLST scheme that would simultaneously potentiate species/subspecies identification and clarification, along with phylogenetic relationships, while an additional per-species accessory genome MLST scheme (integrated within a wgMLST scheme) would increase species-level phylogenetic resolution towards epidemiological inferences. As such, a wgMLST scheme would allow for a dynamic approach that combines both an initial species identification with high-resolution species/subspecies phylogenetic analysis.

The integration of genomic data into clinical microbiology is progressively transforming diagnostic workflows [147,156]. Regardless of its high discriminatory power, the impact of WGS on real-time clinical decision-making in MAC disease cases and epidemiological scenarios remains limited, mainly due to the time required for culture, sequencing, and bioinformatic analysis [134,135,154]. For instance, early initiation of appropriate treatment is associated with improved outcomes; however, delayed access to subspecies-level identification and resistance-associated genomic information could increase the risk of suboptimal antimicrobial therapy, prolong empiric treatment, and contribute to treatment failure [147,154]. In this context, targeted next-generation sequencing (tNGS) emerges as a culture-independent, multiplex PCR-based solution that enables simultaneous amplification and sequencing of predefined genomic regions associated with pathogen identification and antimicrobial resistance (Figure 3). Unlike WGS, tNGS can be applied directly to clinical specimens, offering a faster turnaround time as it is independent of culture isolation. Recent systematic reviews and meta-analyses have demonstrated that tNGS provides high diagnostic performance for drug-resistant *M. tuberculosis* [159,160]. These findings support its potential diagnostic utility as a rapid and broader genotypic drug susceptibility predictor and highlight its role as an emerging approach in routine settings. As several genetic markers have already been proposed in association with resistance (as detailed in Table 3), these could be used as starting points for the development of a tNGS method for resistance screening, with the advent of the loci panel being expanded as the knowledge increases. However, despite promising results in *M. tuberculosis*, the development of the tNGS method for MAC may currently be hampered by the lack of validated discriminatory antimicrobial resistance markers and the complete knowledge of the genetic diversity of circulating MAC strains. These limitations are further compounded by ecological and biological constraints, including the slow-growing nature of MAC, heterogeneous distribution across environmental reservoirs, and difficulties in environmental isolation. In addition, data suggests that MAC lineages are often associated with environmental sources rather than direct transmission and are widely distributed geographically, reinforcing the importance of integrating genomic, environmental, and clinical data in the same framework.

## 9. Conclusions

The global burden of NTM infections, particularly those caused by the MAC, is increasing as a potential emerging public health challenge. However, accurately defining the epidemiology, transmission dynamics, and clinical impact of MAC disease remains difficult due to several interconnected challenges. These include unresolved taxonomic and nomenclature issues within MAC, variability in species- and subspecies-level identification, heterogeneous epidemiological definitions across studies, and the limited availability of harmonized surveillance systems. This underscores the urgent need for strengthened and coordinated surveillance systems to better define the true epidemiology of MAC disease. WGS has emerged as a central tool for high-resolution species identification, outbreak investigation, and improved understanding of transmission dynamics, representing a key advancement in MAC research. However, significant surveillance challenges persist, such as the lack of mandatory notification in many countries, the absence of harmonized typing methodologies, and limited integration of clinical, laboratory, and environmental data. The integration of genomic approaches into routine clinical and public health practice remains uneven, and important gaps persist in capturing the global genetic diversity and population structure of circulating MAC strains. In addition, important knowledge gaps remain regarding the mechanisms underlying treatment failure in MAC disease, like the distinction between genetic antimicrobial resistance and phenotypic tolerance mechanisms, such as biofilm-associated persistence. Improving the understanding of these processes will be important to support the interpretation of susceptibility testing results and the development of more effective treatment strategies. Furthermore, environmental reservoirs further complicate the understanding of transmission pathways, while disparities in laboratory capacity contribute to inconsistent species-level identification across regions. Efforts are still required in order to ensure the capture of the global genetic diversity and subspecies population structure of circulating MAC strains worldwide, as data remains geographically and niche-biased. As such, WGS should target underrepresented subspecies, diverse MAC-associated environmental sources, and sample types, in order to achieve a broader genomic landscape of the complex. Additionally, the development, validation, and implementation of a harmonized and high-resolution genotyping strategy would improve surveillance at the global level. Strengthening this integrated framework combining clinical, microbiological, and environmental data would be essential to improve disease monitoring, enhance comparability across settings, and support more effective public health responses to the growing burden of MAC-related disease.

## 10. Literature Search and Study Selection

The literature was identified through searches in PubMed, Google Scholar, Science Direct, and ResearchGate using combinations of the terms “*Mycobacterium avium* complex”, “MAC”, “nontuberculous mycobacteria”, “epidemiology”, “whole genome sequencing”, “genomics”, “antimicrobial resistance”, and “diagnosis”. The review primarily focused on literature published between 2015 and 2026, although older and historical studies were included when considered essential to contextualize the evolution of MAC taxonomy, epidemiology, and clinical management. Studies were selected based on their relevance to the topics addressed in this review, including epidemiology, clinical manifestations, antimicrobial resistance, and genomic surveillance.

## Figures and Tables

**Table 1:** Typical clinical manifestations associated with human *Mycobacterium avium* complex disease [28,63,73].

Clinical Manifestation	Species
Pulmonary Infection	*M. intracellulare*, *M. chimaera*, MAH
Cystic Fibrosis	*M. chimaera* and MAH
Disseminated Infections	MAC (various species)
Lymphadenitis	MAC (various species)

**Table 2:** Recent epidemiological studies across different countries.

Country	Year	Study Type	Target Dataset	Study Population	Predominant Species	Study Period	Reference
Denmark	2024	Nationwide epidemiological study	Patients with NTM isolation	4123	MAC (62%)—*M. avium* (80%) and *M. intracellulare* (13%)	1991–2022	[85]
Germany	2023	Laboratory-based surveillance study	NTM isolates	11,430	MAC (49.6%)	2016–2020	[86]
India	2025	Systematic review and meta-analysis	Pooled NTM-positive cases from studies of patients with suspicion of NTM infection	67	*M. intracellulare* (32.8%)	2019–2023	[87]
Italy	2025	Retrospective study	NTM isolates	425	MAC (47%)—*M. avium* (28%) and *M. intracellulare* (15%)	2011–2023	[88]
Japan	2025	Nationwide laboratory-based surveillance study	Pulmonary NTM isolates	21,791	*M. avium and M. intracellulare (93%)*	2013–2017	[89]
Mainland China and Taiwan	2025	Systematic review and meta-analysis	Pooled data from 23 studies encompassing NTM-positive isolates	17,959	*M. intracellulare*	2013–2024	[90]
Portugal	2022	Nationwide retrospective laboratory-based study	NTM isolates	1118	MAC (40%)	2014–2020	[23]
UK, Germany, France, Italy, Spain, and Japan	2024	Cross-national study	Patients with NTM isolation	1429	MAC (80%)—79% in Europe and 85% in Japan	2012–2013	[91]
United States of America	2024	Laboratory-based surveillance study	NTM isolates	17,848	MAC (70%)	2019–2022	[92]

Note: Due to differences in study design, surveillance periods, study populations, and population prevalence estimates should not be directly compared across studies. The table is intended to illustrate the predominance of MAC species across diverse epidemiological settings.

**Table 3:** Major antimicrobial resistance mechanisms identified in MAC, including associated genes and resistance phenotypes.

Type of Resistance	Gene/System	Resistance Phenotype	Species	Mechanism	Evidence Type	Reference
Associated resistance	*rrl* (23S rRNA)	Macrolide	MAC	Ribosomal modification prevents macrolide binding	Clinically validated in clinical MAC isolates	[118]
	*rrs* (16S rRNA)	Aminoglycoside	MAC	Ribosomal target modification	Clinically validated in clinical MAC isolates	[119,120]
	*rpoB*	Rifamycin	*M. avium*	RNA polymerase alteration	Resistance-associated in MAC clinical isolates; limited validation compared with *M. tuberculosis*	[121]
Resistance-associated mechanism	*EmbAB*	Associated with reduced ethambutol susceptibility	*M. avium*	Altered arabinosyltransferase activity affecting cell-wall arabinan synthesis	Putative association	[127]
Efflux-mediated resistance/inducible tolerance	*MAV_2510* (*MmpL5/MmpS5*)	Clofazimine/Bedaquiline	MAC	Active drug extrusion	Experimentally validated—in vitro	[125]
	*MAV_3306*	Azithromycin		Putative efflux-associated mechanism contributing to decreased susceptibility		[126]
	*MAV_1406*	Azithromycin/Clarithromycin				
	*MAV_1695*	Bedaquiline				
Tolerance-associated mechanism	Biofilm formation	Increased antimicrobial tolerance/persistence	MAC	Reduced antibiotic penetration, persistence phenotype and metabolic adaptation within biofilms	Phenotypic association (GPL-associated matrix)–in vitro	[48,128]
Intrinsic resistance	Lipid-rich cell wall	Broad intrinsic reduced susceptibility	MAC	A hydrophobic barrier limiting drug penetration	Established biological mechanism	[129]

## Data Availability

No new data were created or analyzed in this study. Data sharing is not applicable to this article.
